# A High Frequency of Circulating Th22 and Th17 Cells in Patients with New Onset Graves’ Disease

**DOI:** 10.1371/journal.pone.0068446

**Published:** 2013-07-11

**Authors:** Di Peng, Bingchuan Xu, Ye Wang, Hui Guo, Yanfang Jiang

**Affiliations:** 1 Department of Central Laboratory, the Second Part of the First Hospital, Jilin University, Changchun, China; 2 Key Laboratory of Zoonosis Research, Ministry of Education, Institute of Zoonosis, Jilin University, Changchun, China; Northwestern University Feinberg School of Medicine, United States of America

## Abstract

T-helper (Th) 22 and Th17 cells are involved in the pathogenesis of autoimmune diseases. However, their roles in the pathogenesis of Graves’disease (GD) are unclear. This study is aimed at examining the frequency of peripheral blood Th22, Th17, and Th1 cells and the levels of plasma IL-22, IL-17, and IFN-γ in patients with GD. A total of 27 patients with new onset GD and 27 gender- and age-matched healthy controls (HC) were examined for the frequency of peripheral blood Th22, Th17, and IFN-γ cells by flow cytometry. The concentrations of plasma IL-22, IL-17, and IFN-γ were examined by enzyme-linked immunosorbent assay. The levels of serum TSHR antibodies (A-TSHR), free triiodothyronine (FT3), free thyroxine (FT4), and thyroid stimulating hormone (TSH) were examined by radioimmunoassay and chemiluminescent assay, respectively. The levels of serum TSAb were examined by enzyme-linked immunosorbent assay. In comparison with those in the HC, significantly elevated percentages of Th22 and Th17 cells, but not Th1 cells, and increased levels of plasma IL-22 and IL-17, but not IFN-γ, were detected in GD patients (P<0.0001, for both). The percentages of both Th22 and Th17 cells and the levels of plasma IL-22 and IL-17 were correlated positively with the levels of serum TSAb in GD patients (r = 0.7944, P<0.0001; r = 0.8110, P<0.0001; r = 0.7101, p<0.0001; r = 0.7407, p<0.0001, respectively). Th22 and Th17 cells may contribute to the pathogenesis of GD.

## Introduction

Graves’ disease (GD) is an organ-specific autoimmune disease that is attributed to overstimulation of the thyroid glands by agonistic anti-thyrotropin receptor antibody (thyroid-stimulating antibody; TSAb), leading to hyperthyroidism and thyroid enlargement [1], [2]. GD occurs predominantly in women and its incidence is approximately 0.25–1.09% in the Chinese population [3]. GD represents both the most common cause of, hyperthyroidism and an archetypical example of antibody-mediated organ-specific autoimmunity. The pathogenesis of GD is complex and heterogeneous, and its etiology remains unclear. Since TSAb is a hallmark of GD T helper type 2 (Th2) responses have been associated with the pathogenesis of GD. Strikingly, recent studies have suggested that other types of functional T cells, such as Th17 cells, also play an important role in the pathogenesis of GD [4]–[7]. However, there is little information available about the role of other types of immunocompetent cells in the development and progression of GD.

Antigen determinants activate naïve CD4+ T cells, which can differentiate into Th17 and Th22 cells (besides Th1 and Th2 cells), which are regulated by RORγt and aryl hydrocarbon receptor transcription factor, respectively [8], [9]. Th17 cells predominantly produce IL-17A, while Th22 cells secrete IL-22 [10]. Both IL-17A and IL-22 have been shown to be pro-inflammatory cytokines that participate in the pathogenesis of autoimmune diseases, such as rheumatoid arthritis (RA) [11], Crohn’s disease [12], systemic lupus erythematosus (SLE) [13], and psoriasis [14]. A previous study has shown that a high frequency of Th17 cells and high levels of IL-17 are present in patients with severe GD [4] and that Th17, together with Th1 cells, may contribute to the development of Hashimoto’s thyroiditis [15]. However, there is little information about whether higher frequency of Th17 and higher concentrations of IL-17A also exist in Chinese patients with GD and how Th17 responses are associated with the concentrations of TSAb and thyroid function in GD patients. Furthermore, it is unclear whether Th22 and IL-22 responses are associated with the development of GD. In addition, IL-22 and IL-17 can be secreted by some subsets of CD4^+^ T cells [4], [9]. However, what the levels of these cytokines are in GD patients and how they are related to the thyroid function have not been explored.

In this study, we characterized the frequency of peripheral blood Th22, Th17, and Th1 cells by flow cytometry and measured the concentrations of plasma IL-22, IL-17, and IFN-γ by enzyme-linked immunosorbent assay (ELISA) in 27 Chinese patients with new onset GD. Furthermore, we analyzed the potential association of the percentages of Th22, Th17, and Th1 cells with the clinical measures in these GD patients. Our findings indicated that higher percentages of Th22 and Th17 cells were associated with higher concentrations of TSAb in Chinese patients with new onset GD.

## Results

### A Higher Frequency of IL-17A+ and IL-22+ CD4+ T Cells in GD Patients

To determine the frequency of different subsets of functional CD4+ T cells, a total of 27 Chinese patients with new onset GD and 27 gender- and age-matched HC were recruited. As expected, there was no significant difference in the distribution of age and gender and in the WBC and lymphocyte counts between these two groups (Table 1). Furthermore, the concentrations of serum FT3 and FT4 in GD patients were significantly higher than that in the HC, while the concentrations of serum TSH in the GD patients were significantly lower than that in the HC. In addition, the patients had abnormally higher levels of A-TSHR. Collectively, these GD patients had hyperthyroidism and abnormal levels of thyroid-specific antibodies.

**Table 1:** The demographic and clinical characteristics of the participants.

	Healthy controls (n = 27)	GD patients (n = 27)
Gender (M/F)	2/25	4/23
Age (year) median(range)	44 (30–56)	41 (13–59)
TSH (µIU/mL)	2.2 (0.3–3.3)	0.01 (0–0.022)*
FT4 (pmol/L)	15.39 (12.89–17.21)	65.37 (17.38–126.33)*
FT3 (pmol/L)	4.42 (3.50–5.60)	30.80 (6.5–38.22)*
A-TSHR(IU/mL)	0.64 (0.38–0.94)	17.30 (3.71–40.00)*
TsAb (ng/mL)	7.19 (6.14–8.37)	41.48(38.50–44.55)
WBC (×10^9^/L)	5.51 (4.78–6.83)	5.98 (4.01–8.20)
Lymphocytes (×10^9^/L)	2.70 (1.4–3.4)	2.70 (1.1–3.6)

Data shown are the case number or median (range). The normal ranges are 0.3–3.6 pmol/L for TSH; 3.5–6.5 pmol/L for FT3; 11.5–22.7 pmol/L for FT4; 0.3–1.22 IU/L for A-TSHR; 4.0–10.0×10^9^/L for WBC and 1.2–3.4×10^9^/L for Lymphocytes. *p<0.05 vs. the healthy controls.

**Figure 1:**
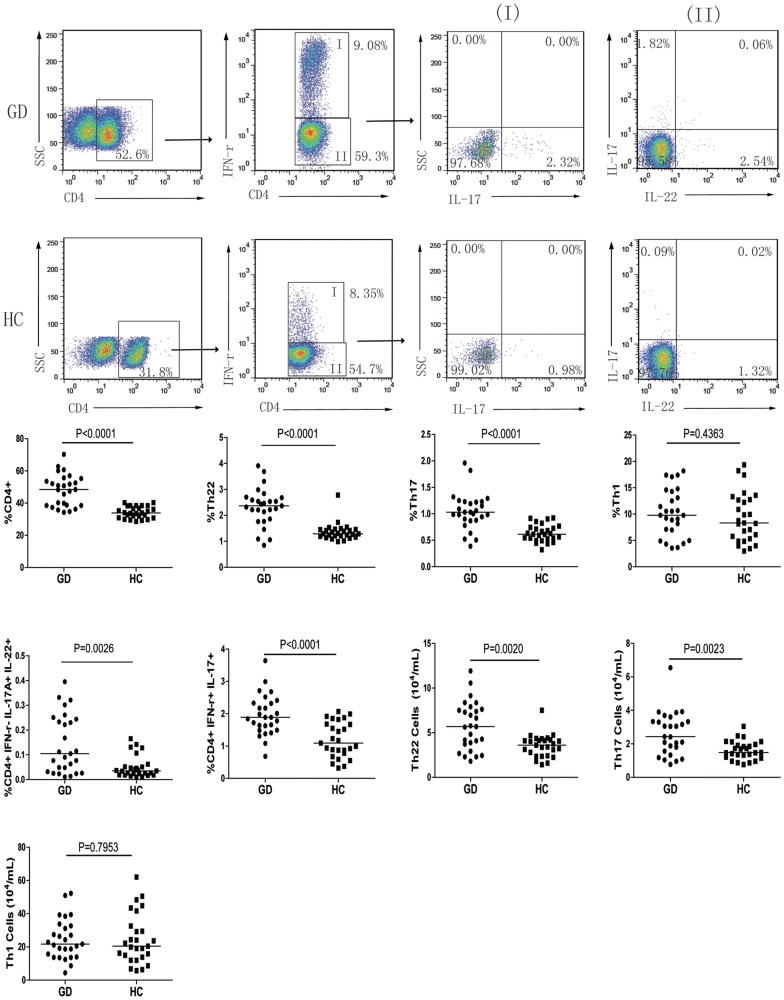
Flow cytometry analysis of different subsets of CD4+ T cells. PBMCs were isolated form individual participants and stimulated with, or without, PMA/ionomycin and harvested. The cells were stained with APC-anti-CD4, fixed, and permeabilized, followed by intracellular staining with FITC-anti-IL-17, PE-Cy7-anti-IFNγ, and PE-anti-IL-22 and flow cytometry. Subsequently, the cells were gated first on CD4^+^ cells for analysis of the frequency of CD4^+^IFNγ^+^ and CD4^+^IFNγ^−^ cells. The CD4^+^IFNγ^+^ cells were further analyzed for CD4^+^IFNγ^+^IL-17^+^ cells (column I), while the CD4^+^IFNγ^−^ cells were further analyzed for CD4^+^IFNγ^−^IL-17^+^, CD4^+^IFNγ^−^IL-22^+^, and CD4^+^IFNγ^−^IL-17^+^IL-22^+^ cells (column II), followed by quantitative analyses. Data are representative charts or expressed as the mean values of individual participants from sequential experiments. A. Representative charts of flow cytometry analysis; B. Quantitative analysis.

We analyzed the percentages of circulating CD4^+^ T cells in 27 patients with new onset GD and 27 gender- and age-matched healthy controls (HC), and we found that the percentages of CD4^+^ T cells in the GD patients were significantly higher than that in the HC (p<0.0001, Fig. 1). To characterize the frequency of the different subsets of CD4^+^ T cells, PBMCs were isolated from individual participants and stimulated by PMA/ionomycin, followed by intracellular staining with anti-cytokine antibodies and flow cytometry analysis. Following gating on CD4^+^ T cells, there was no significant difference in the frequency of CD4^+^IFN-γ^+^IL-17A^−^IL-22^−^ Th1 cells in total CD4^+^ T cells between these two groups of subjects (Fig. 1). In contrast, the percentages of CD4^+^IFN-γ^–^IL17A^+^IL-22^−^ Th17, CD4^+^IFN-γ^–^IL17A^+^IL-22^+^ Th17/Th22, CD4^+^IFN-γ^–^IL17A^–^IL-22^+^ Th22, and CD4^+^IFN-γ^+^IL17^+^ Th1/Th17 cells in total CD4^+^ T cells in the GD patients were significantly higher than that in the HC (P<0.0001, P = 0.0026, P<0.0001, and P<0.0001, respectively). We further analyzed the total numbers of Th22, Th17 and Th1 cells, and we found the total numbers of Th22 and Th17 cells in the GD patients were significantly higher than that in the HC (Fig. 1, P = 0.0020 and P = 0.0023, respectively). There was no significant difference in the total numbers of Th1 cells between these two groups of subjects (Fig. 1, P = 0.7953).

**Figure 2:**
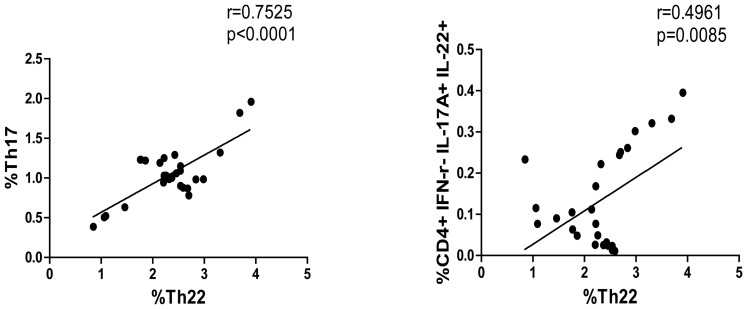
The correlation between the percentages of Th17, Th22 and Th17/Th22 cells in patients. The potential correlations among different subsets of CD4+ T cells were analyzed by Spearman rank correlation test. Data shown are the mean values of individual patients (n = 27). There was no significant correlation between the percentages of Th17 and Th17/Th22 cells in those patients.

To investigate the relationship between different subsets of CD4^+^ T cells, we found that the percentages of Th22 cells were correlated positively with the percentages of Th17 in GD patients and that the percentages of Th22 cells, but not Th17 cells, were correlated positively with the percentages of Th17/Th22 cells in GD patients (r = 0.7525, p<0.0001; r = 0.4961, p = 0.0085, respectively, Fig. 2). However, there was no other significant correlation among these subsets of CD4^+^ T cells in those patients (data not shown). Hence, a higher frequency of IL-17A^+^ and IL-22^+^ CD4^+^ T cells was present in Chinese patients with new onset GD.

### Higher Concentrations of Serum IL-17 and IL-22 are Present in GD Patients

**Figure 3:**
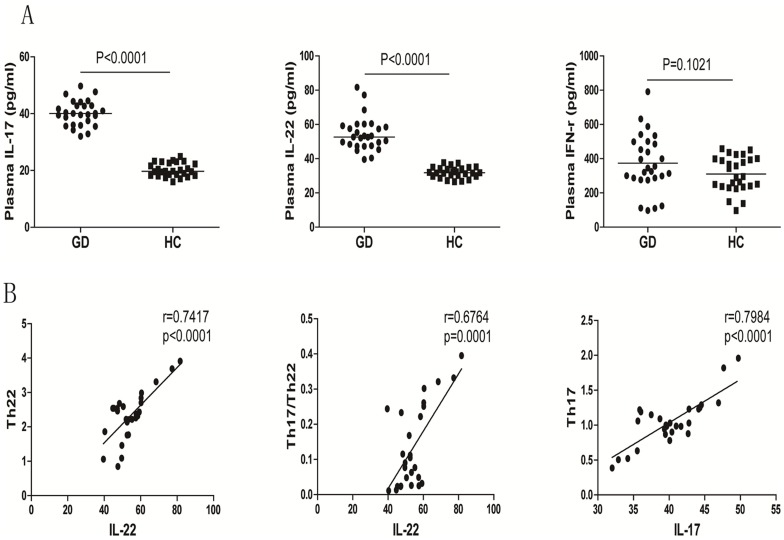
The concentrations of plasma cytokines in the subjects. The concentrations of plasma IL-17, IL-22, and IFNγ in individual subjects were measured by ELISA and the potential association of the concentrations of plasma cytokines with the percentages of corresponding cells was analyzed by Spearman rank correlation test. Data are expressed as the mean values of individual subjects (n = 27 for each group) from five separate experiments. A. ELISA analysis of the levels of plasma cytokines; B. Correlation analysis. The concentrations of plasma IL-17 were not correlated significantly with the percentages of Th17/Th22 cells in those patients (data not shown).

To test the function of different subsets of CD4^+^ T cells, we measured the concentrations of plasma IL-22 and IL-17 in individual participants by ELISA. We found that the concentrations of plasma IFN-γ in the GD patients were only slightly higher than that in the HC (p = 0.1021). In contrast, the concentrations of plasma IL-22 and IL-17 in the GD patients were significantly higher than that in the HC (p<0.0001 for both, Fig. 3A). Further analysis revealed that the concentrations of plasma IL-22 were correlated positively with the percentages of Th22 and Th17/Th22 cells (r = 0.7417, p<0.0001; r = 0.6764, p = 0.0001, respectively, Fig. 3B) and that the concentrations of plasma IL-17 were correlated positively with the percentages to Th17 cells (r = 0.7984, p<0.0001 Fig. 3B), but not Th17/Th22 cells (data not shown), in the GD patients. Thus, Th17 and Th22 cells had high function in the GD patients.

### The Percentages of Peripheral Blood Th22 and Th17 Cells are Positively Correlated with the Concentrations of Serum TSAb in GD Patients

**Figure 4:**
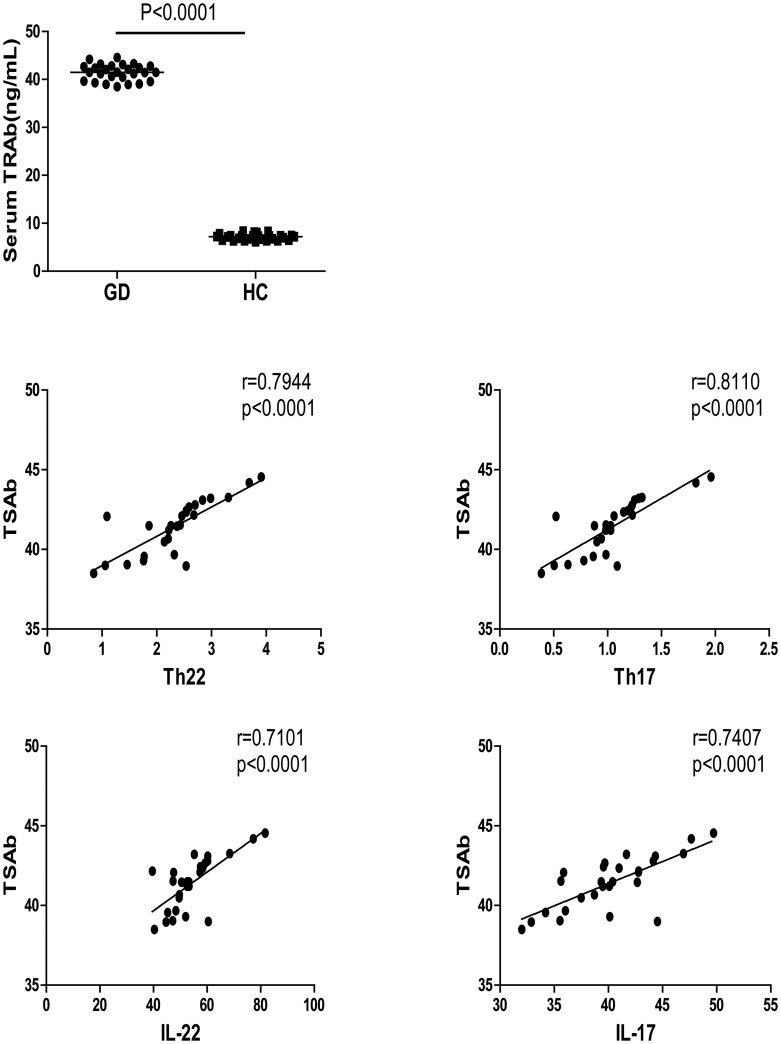
The concentrations of serum TSAb in the subjects. The concentrations of serum TSAb in individual subjects were measured by ELISA, and the potential correlations between the levels of serum TSAb and the percentages of Th22, Th17 cells, and the levels of serum IL-17 and IL-22 in the GD patients were analyzed by Spearman rank correlation test. Data are expressed as the mean values of individual patients (n = 27). There was no significant association between the percentages of Th17, Th22, or the levels of plasma IL-17 and IL-22 and the levels of serum FT3, FT4, and TSH in those patients (data not shown).

The production of agonistic TSAb that over-stimulates the production of thyroid hormones is a pathogenic hallmark of GD. We further analyzed the relationships between different subsets of CD4^+^ T cells and the concentrations of TSH, FT3, FT4, and TSAb in GD patients. We found that the percentages of peripheral blood Th17 and Th22 cells were correlated positively with the concentrations of serum TSAb in the GD patients (r = 0.7944, p<0.0001; r = 0.8110, p<0.0001, respectively, Fig. 4). However, there was no significant association of the frequency of Th17 and Th22 cells with the concentrations of serum FT3, FT4, and TSH in those patients (data not shown). Further analysis revealed that the concentrations of plasma IL-17 and IL-22 were also correlated positively with the levels of serum TSAb in those patients (r = 0.7101, p<0.0001; r = 0.7407, p<0.0001, respectively, Fig. 4). Therefore, increased numbers of Th22 and Th17 cells are associated with the development of GD in Chinese patients.

## Discussion

Th17 cells secrete IL-17A and participate in the pathogenesis of many autoimmune diseases [16], [17]. Th22 cells produce IL-22 and are crucial regulators of tissue remodeling as well as epidermal immunity [18]. In this study, we examined the frequency of peripheral blood Th1, Th17, and Th22 cells and the concentrations of plasma IFNγ, IL-17A, and IL-22 in 27 GD patients and 27 HC. We found that the percentages of peripheral blood Th17 and Th22 as well as CD4^+^IL-17^+^IL-22^+^ Th17/Th22 cells, but not Th1 cells, were significantly higher in the patients than that in the HC. Consistently, the concentrations of plasma IL-17A and IL-22, but not IFNγ in the patients were significantly higher than that in the HC. More importantly, the percentages of both Th22 and Th17 cells and the levels of plasma IL-22 and IL-17 were correlated positively with the levels of serum TSAb, but not with the levels of FT3, FT4, and TSH in GD patients. Given that the abnormal levels of TSAb are responsible for the development of GD [19], our novel findings suggest that a higher frequency of Th17 and Th22, as well as higher levels of IL-17A and IL-22, may contribute to the pathogenesis of GD in Chinese patients. To the best of our knowledge, this is the first report about the higher frequency of Th22 and higher levels of plasma IL-22 in GD patients. Our findings may provide new insights into the pathogenesis of GD. Conceivably, Th17 and Th22 cells are new targets for the design of immunotherapies for the intervention of GD.

Th17 cells play important roles in the pathogenic process of some autoimmune diseases. Horie et al. [20] indicated the important role of Th17 cells in the pathogenesis of Graves’ hyperthyroidism in a mouse model. We found that the percentages of peripheral blood Th17 cells in GD patients were significantly higher than that in the HC, consistent with a previous observation in a Japanese population [4]. However, our findings were in disagreement with that found in Hispanic GD patients, who displayed very low frequencies of peripheral blood Th17 cells [21]. The difference may stem from the different populations of patients studied. Indeed, the role of Th17 cells in the pathogenesis of Graves’ hyperthyroidism depends on different genetic backgrounds of mice [20]. In addition, we also detected significantly higher levels of plasma IL-17 in the patients, consistent with a previous study [22]. Moreover, we found that the concentrations of plasma IL-17 were correlated positively with the percentages of peripheral blood Th17 cells in the GD patients. These suggest that IL-17 is predominantly secreted by Th17 cells in those patients and that the increased numbers of Th17 cells and higher levels of IL-17 may participate in the pathogenesis of GD in Chinese patients. We are interested in further investigating how Th17 cells regulate pathogenic B cell activation and TSAb production, contributing to the pathogenesis of GD.

Previous studies have shown that CD4^+^IFN-γ^+^IL-17^+^ Th1/Th17 cells are a rare population, but are significantly higher in RA patients, particularly in the synovial fluid of RA patients [23], [24]. The development of Th1 and Th17 cells depends on the cytokine environment [25], [26]. In addition, IL-17 and IFN-γ can be detected in human memory CD4+CD45+RO+ T cells [27]. We found that the percentages of Th1/Th17 cells in the GD patients were significantly higher than that in the HC. However, we did not detect any association of the percentages of Th1/Th17 cells with the frequency of Th1, Th17 cells, or the concentrations of plasma IFNγ or IL-17 in those patients. In addition, there was no significant correlation between the percentages of Th1/Th17 cells and the levels of serum TSAb in those patients. These data suggest that Th1/Th17 cells may not be potent effectors, contributing to the development of GD. Given that, following activation, T cells can differentiate into different functional T cells, these Th1/Th17 cells may be early differentiated and uncommitted cells.

Th22 cells are a unique subset of T cells and regulate tissue modeling and epidermal immunity [18]. We found that the percentages of peripheral blood Th22 and the concentrations of plasma IL-22 in the patients were significantly higher than those in the HC. Furthermore, the percentages of Th22 cells were correlated with the levels of plasma IL-22 in those patients, suggesting that Th22 cells are major producers of IL-22 in the GD patients. Indeed, previous studies have detected significantly higher percentages of peripheral blood Th22 cells in patients with ankylosing spondylitis (AS), RA, SLE, and psoriasis [28]–[30]. Our findings extend previous observations and support the notion that Th22 cells are not only involved in epidermal immunity, but also regulate other organ-specific autoimmunity in humans. It is notable that we detected a significantly higher frequency of CD4^+^Th17^+^IL-22^+^ Th17/Th22 cells in the GD patients, as compared with that in the HC, and that the percentages of Th17/Th22 cells were correlated with the levels of plasma IL-22, but not IL-17 in those patients. Given that Th17 and Th22 development are positively regulated by IL-6, but negatively by TGFβ1 [31], it is possible that Th17/Th22 cells are early activated and uncommitted T cells in an inflammatory environment, where high levels of IL-6, but low levels of TGFβ1 are present. These cells may further differentiate into either Th22 or Th17 cells. Indeed, a previous study has shown a significantly lower frequency of TGFβ1-producing CD4+CD25+FOXP3+ Treg cells in GD patients [32]. We are interested in further investigating how these cytokines regulate Th17/Th22 cell development and their function during the pathogenic process of GD.

If confirmed, our findings may provide new insights into understanding the pathogenesis of GD and aid in developing new measures for early diagnosis and predicting patient outcomes after anti-thyroid drug therapy.

### Conclusion

We found significantly higher percentages of peripheral blood Th17, Th22, Th1/Th17, and Th17/Th22, but not Th1 cells, and higher levels of plasma IL-17 and IL-22 in GD patients. The percentages of Th17, Th22, and the levels of plasma IL-17 and IL-22 were correlated positively with the levels of serum TSAb in those patients, suggesting that Th17 and Th22 cells and their cytokines may contribute to the pathogenesis of GD in Chinese. We recognize that our studies had limitations, including a small sample size, one time point, and the lack of a functional study of these different subsets of T cells in those patients. Therefore, further studies in a bigger population are needed to validate these findings and to determine the role of Th17 and Th22 in the pathogenesis of GD.

## Materials and Methods

### Study Subjects

Twenty seven patients with new onset GD were recruited from the outpatient service of the First Hospital of Jilin University, Changchun, China between December 2011 and November 2012. Individual patients with GD were diagnosed according to the clinical evidence of hyperthyroidism (thyroid stimulating hormone, TSH <0.3 uIU/L, normal range: 0.3–3.6 pmol/L; free triiodothyronine (FT3) >6.5 pmol/L, normal range: 3.5–6.5 pmol/L; and free thyroxine (FT4) >22.7 pmol/L, normal range: 11.5–22.7 pmol/L), the presence of anti-TSHR antibodies (normal range: 0.3–1.22 IU/L), and diffuse goiter, as investigated by ultrasound examination. Twenty seven gender- and age-matched healthy volunteers without a family history of GD were recruited from the outpatient service of the same hospital and served as controls. Individual participants were excluded if she/he had current or a history of other autoimmune diseases, such as type 1 diabetes (T1D), RA, SLE, multiple sclerosis and autoimmune hepatitis, or other chronic inflammatory diseases, such as metabolic syndrome, inflammatory bowel disease, and chronic renal disease. Written informed consent was obtained from all participants, and the study was approved by the Institutional Ethics Board of School of Medicine, Jilin University. The demographic and clinical data of individual participants were collected from hospital records and reviewed by endocrinologists. Their demographic and clinical characteristics are summarized in Table 1.

### Laboratory Tests

Venous blood samples were obtained from individual participants and subjected to routine laboratory tests for full blood counts and serum chemistry using a Hitachi 7600 automatic analyzer (Hitachi High Technologies, Tokyo, Japan). The concentrations of serum TSH, FT3, and FT4 in individual participants were examined by chemiluminescent enzyme immunoassay using specific kits (Ortho-Clinical Diagnostics, Raritan, NJ, USA). The concentrations of serum anti-TSH receptor antibodies (A-TSHR) in all participants were examined by radioimmunoassay using the specific kit, according to the manufacturers’ instruction (Cosmic, Tokyo, Japan).

### Isolation and Stimulation of PBMCs

Individual participants fasted overnight, and their peripheral venous blood samples were collected. Peripheral blood mononuclear cells (PBMCs) were isolated by density-gradient centrifugation using Ficoll-Paque Plus (Amersham Biosciences, Little Chalfont, UK). PBMCs (10^6^/mL) were stimulated in duplicate with 50 ng/mL of phorbol myristate acetate (PMA) and 1.0 µg/mL of ionomycin (Sigma, St. Louis, MO, USA) in 10% human AB type of sera in RPMI 1640 medium at 37°C in a humidified incubator with 95% air and 5% carbon dioxide for 2 hours and then cultured for another 4 hours in the presence of 0.5 µg/mL of brefeldin A (BFA, Sigma). The control PBMCs were cultured in medium alone.

### Flow Cytometry Analysis

The frequency of CD4^+^IFN-γ^–^IL17A^–^IL-22^+^ Th22, CD4^+^IFN-γ^–^IL17A^+^IL-22^−^ Th17, CD4^+^IFN-γ^–^IL17A^+^IL-22^+^ Th17/Th22, CD4^+^IFN-γ^+^IL-17A^−^IL-22^−^ Th1, and CD4^+^ IFN-γ^+^IL17^+^ Th1/Th17 cells in individual samples were determined by flow cytometry following intracellular staining with anti-cytokine antibodies. Briefly, the stimulated PBMCs were harvested and stained with allophycocyanin (APC)-labeled anti-CD4, fixed with the Perm/Fix solution, and permeabilized, followed by staining with fluorescein isothiocyanate (FITC)-labeled anti–IL-17, PE-Cy7-labeled anti-IFNγ (Becton Dickinson, San Diego, USA), and PE-labeled anti–IL-22 (R&D Systems, Minneapolis, MN, USA). Subsequently, the cells were analyzed on a FACSCalibur (BD Biosciences, San Jose, USA) using FlowJo 7.6.2 software.

### Enzyme-linked Immunosorbent Assay (ELISA)

The concentrations of plasma IFN-γ, IL-17 and IL-22 in individual participants were determined by ELISA using specific cytokine kits, according to the manufacturers’ instruction (R&D Systems). The concentrations of serum TSAb in individual participants were determined by ELISA using a specific kit, according to the manufacturers’ instruction (Biological Experimental Factory of Tianjin Medical University, China). The limitation of detection for IFN-γ, IL-17, and IL-22 was 9.4 pg/mL, 31.2 pg/mL, or 15.6 pg/mL, respectively. The limitation of detection for TSAb was 0.59 ng/mL.

### Statistical Analysis

All data are expressed as individual mean or median values and range of each group of the subjects. The difference between two groups was analyzed by the Kruskal-Wallis H nonparametric test. The potential correlations between variables were evaluated by the Spearman rank correlation test using SPSS 19.0 for Windows (SPSS, Chicago, IL, USA). A two-sided P value of <0.05 was considered statistically significant.
